# Supplementary material for: Meta-Analysis Examining the Importance of Creatine Ingestion Strategies on Lean Tissue Mass and Strength in Older Adults
Source: Nutrients. 2021 Jun 2;13(6):1912. doi: 10.3390/nu13061912 (PMC8229907; doi:10.3390/nu13061912)
Supplement: Supplementary file 1 [file nutrients-13-01912-s001.zip › nutrients-1225760-supplementary.pdf]

Supplementary File

Study or Subgroup	Creatine Mean	Creatine SD	Creatine Total	Placebo Mean	Placebo SD	Placebo Total	Weight	Mean Difference IV, Fixed, 95% CI
Aguiar 2013	1.1	1.5	9	0	2.5	9	0.0%	1.10 [-0.80, 3.00]
Bernat 2019	1.21	1.61	12	1.19	1.96	12	21.3%	0.02 [-1.42, 1.46]
Brose 2003 (men)	1.4	4.6	5	0	3.5	2	0.0%	1.40 [-4.91, 7.71]
Brose 2003 (women)	2	2.5	5	0.6	1.2	5	0.0%	1.40 [-1.03, 3.83]
Candow 2008	2.1	1.4	13	0.6	2.1	12	22.0%	1.50 [0.09, 2.91]
Candow 2015 (Cr after)	3	1.9	12	0.5	2.1	12	17.1%	2.50 [0.90, 4.10]
Candow 2015 (Cr before)	1.8	2	15	0.5	2.1	12	18.0%	1.30 [-0.26, 2.86]
Candow 2020	-0.93	5.3	18	-1.89	6.2	20	3.3%	0.96 [-2.70, 4.62]
Chilibeck 2015	-1	2.9	15	-1.3	2.2	18	13.7%	0.30 [-1.49, 2.09]
Chrusch 2001	3.3	4.2	16	1.3	3.2	14	0.0%	2.00 [-0.65, 4.65]
Cooke 2014	2	4.8	10	0.5	6.9	10	0.0%	1.50 [-3.71, 6.71]
Deacon 2008	1.1	2.5	38	0.7	2	42	0.0%	0.40 [-0.60, 1.40]
Eijnde 2003	0.9	4.2	23	0.3	3.5	23	0.0%	0.60 [-1.63, 2.83]
Eliot 2008	2.5	4.6	10	-0.4	7.8	10	0.0%	2.90 [-2.71, 8.51]
Gualano 2011	3	1.3	13	0	1.3	12	0.0%	3.00 [1.98, 4.02]
Johannsmeyer 2016	2.8	4.3	14	0.9	4.3	17	4.7%	1.90 [-1.14, 4.94]
Neves 2011	1.1	3.9	13	0.8	3	11	0.0%	0.30 [-2.46, 3.06]
Pinto 2016	1.8	1.3	13	0.6	1.3	14	0.0%	1.20 [0.22, 2.18]
**Total (95% CI)**			**99**			**103**	**100.0%**	**1.16 [0.49, 1.82]**

Heterogeneity: Chi² = 6.49, df = 6 (P = 0.37); I² = 8%
Test for overall effect: Z = 3.43 (P = 0.0006)

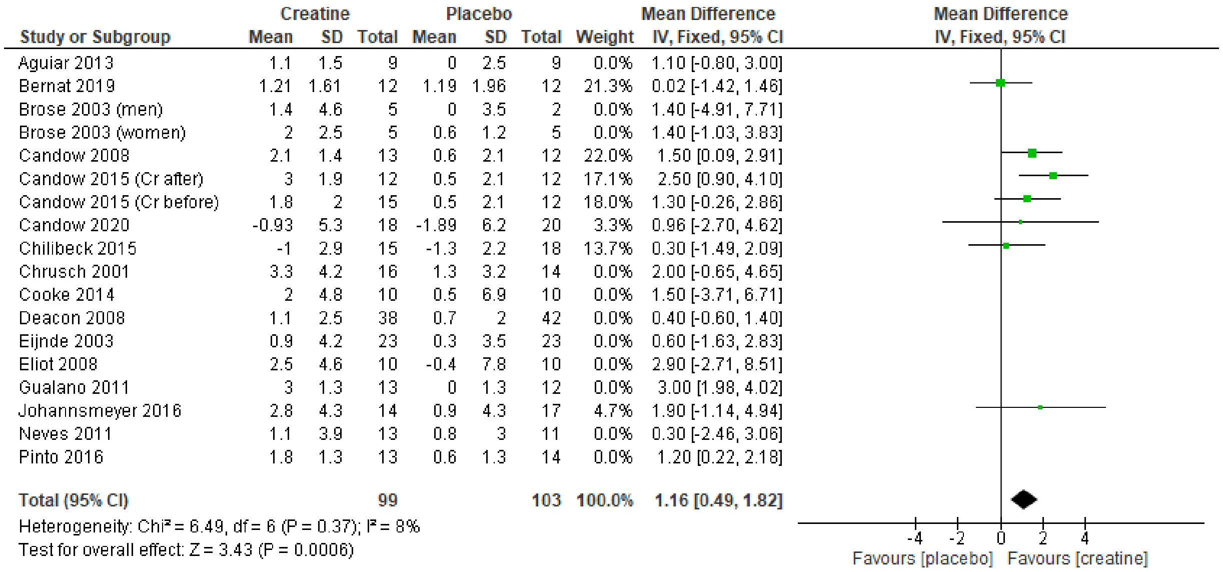

Figure S1: Forest plot for lean tissue mass with the inclusion of only high dose studies that did not include a creatine loading phase.

Study or Subgroup	Creatine Mean	Creatine SD	Creatine Total	Placebo Mean	Placebo SD	Placebo Total	Weight	Mean Difference IV, Fixed, 95% CI
Aguiar 2013	1.1	1.5	9	0	2.5	9	10.3%	1.10 [-0.80, 3.00]
Bernat 2019	1.21	1.61	12	1.19	1.96	12	0.0%	0.02 [-1.42, 1.46]
Brose 2003 (men)	1.4	4.6	5	0	3.5	2	0.9%	1.40 [-4.91, 7.71]
Brose 2003 (women)	2	2.5	5	0.6	1.2	5	6.3%	1.40 [-1.03, 3.83]
Candow 2008	2.1	1.4	13	0.6	2.1	12	0.0%	1.50 [0.09, 2.91]
Candow 2015 (Cr after)	3	1.9	12	0.5	2.1	12	0.0%	2.50 [0.90, 4.10]
Candow 2015 (Cr before)	1.8	2	15	0.5	2.1	12	0.0%	1.30 [-0.26, 2.86]
Candow 2020	-0.93	5.3	18	-1.89	6.2	20	0.0%	0.96 [-2.70, 4.62]
Chilibeck 2015	-1	2.9	15	-1.3	2.2	18	0.0%	0.30 [-1.49, 2.09]
Chrusch 2001	3.3	4.2	16	1.3	3.2	14	0.0%	2.00 [-0.65, 4.65]
Cooke 2014	2	4.8	10	0.5	6.9	10	0.0%	1.50 [-3.71, 6.71]
Deacon 2008	1.1	2.5	38	0.7	2	42	0.0%	0.40 [-0.60, 1.40]
Eijnde 2003	0.9	4.2	23	0.3	3.5	23	7.5%	0.60 [-1.63, 2.83]
Eliot 2008	2.5	4.6	10	-0.4	7.8	10	0.0%	2.90 [-2.71, 8.51]
Gualano 2011	3	1.3	13	0	1.3	12	36.0%	3.00 [1.98, 4.02]
Johannsmeyer 2016	2.8	4.3	14	0.9	4.3	17	0.0%	1.90 [-1.14, 4.94]
Neves 2011	1.1	3.9	13	0.8	3	11	0.0%	0.30 [-2.46, 3.06]
Pinto 2016	1.8	1.3	13	0.6	1.3	14	38.9%	1.20 [0.22, 2.18]
**Total (95% CI)**			**68**			**65**	**100.0%**	**1.81 [1.20, 2.42]**

Heterogeneity: Chi² = 8.50, df = 5 (P = 0.13); I² = 41%
Test for overall effect: Z = 5.79 (P < 0.00001)

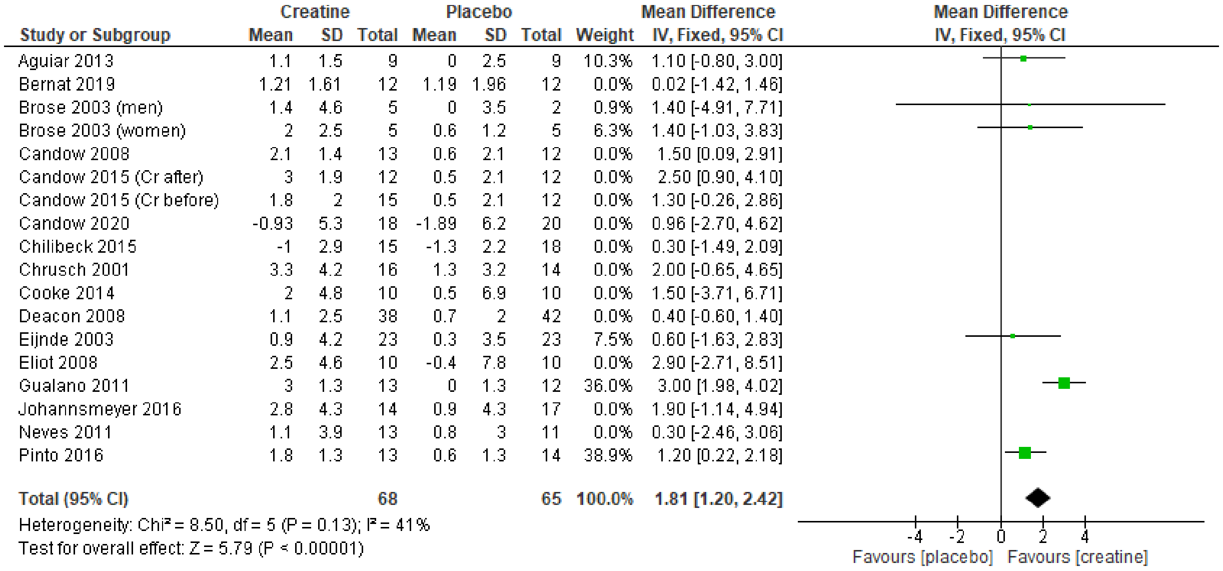

Figure S2: Forest plot for lean tissue mass with the inclusion of only low dose studies that did not include a creatine loading phase.

Study or Subgroup	Creatine Mean	Creatine SD	Creatine Total	Placebo Mean	Placebo SD	Placebo Total	Weight	Std. Mean Difference IV, Fixed, 95% CI
Aguiar 2013	14	17	9	9	5	9	0.0%	0.38 [-0.55, 1.31]
Alves 2013	3.4	4	12	2.7	2.2	10	0.0%	0.20 [-0.64, 1.05]
Bemben 2010	42	13	10	33	6	10	0.0%	0.85 [-0.07, 1.78]
Bermon 1998	1	1	8	0.6	0.4	8	0.0%	0.50 [-0.50, 1.50]
Bernat 2019	19	12.9	12	17.58	7	12	12.4%	0.13 [-0.67, 0.93]
Brose 2003 (men)	30	20	7	22	12	7	0.0%	0.45 [-0.61, 1.52]
Brose 2003 (women)	15	14	6	13	7	6	0.0%	0.17 [-0.97, 1.30]
Candow 2008	11	11	13	9	14	12	12.9%	0.15 [-0.63, 0.94]
Candow 2015 (Cr after)	16	13	12	2	15	12	10.9%	0.96 [0.11, 1.82]
Candow 2015 (Cr before)	15	13	15	2	15	12	12.4%	0.91 [0.10, 1.71]
Candow 2020	11	20.9	18	22	21	20	19.0%	-0.51 [-1.16, 0.13]
Chilibeck 2015	18	15	15	11	14	18	16.5%	0.47 [-0.22, 1.17]
Chrusch 2001	19	13	16	16	11	14		Not estimable
Cooke 2014	8	9	10	7	13	10		Not estimable
Gualano 2011	8	16	13	13	8	12	0.0%	-0.38 [-1.17, 0.42]
Gualano 2014	2.6	4.3	15	1.8	4.9	15		Not estimable
Hass 2007	0.2	0.07	10	0.1	0.06	10	0.0%	1.47 [0.46, 2.48]
Johannsmeyer 2016	15	20	14	13	22	17	15.9%	0.09 [-0.62, 0.80]
Pinto 2016	12	5	13	11	6	14	0.0%	0.17 [-0.58, 0.93]
**Total (95% CI)**			**99**			**103**	**100.0%**	**0.25 [-0.03, 0.53]**

Heterogeneity: Chi² = 11.30, df = 6 (P = 0.08); I² = 47%
Test for overall effect: Z = 1.73 (P = 0.08)

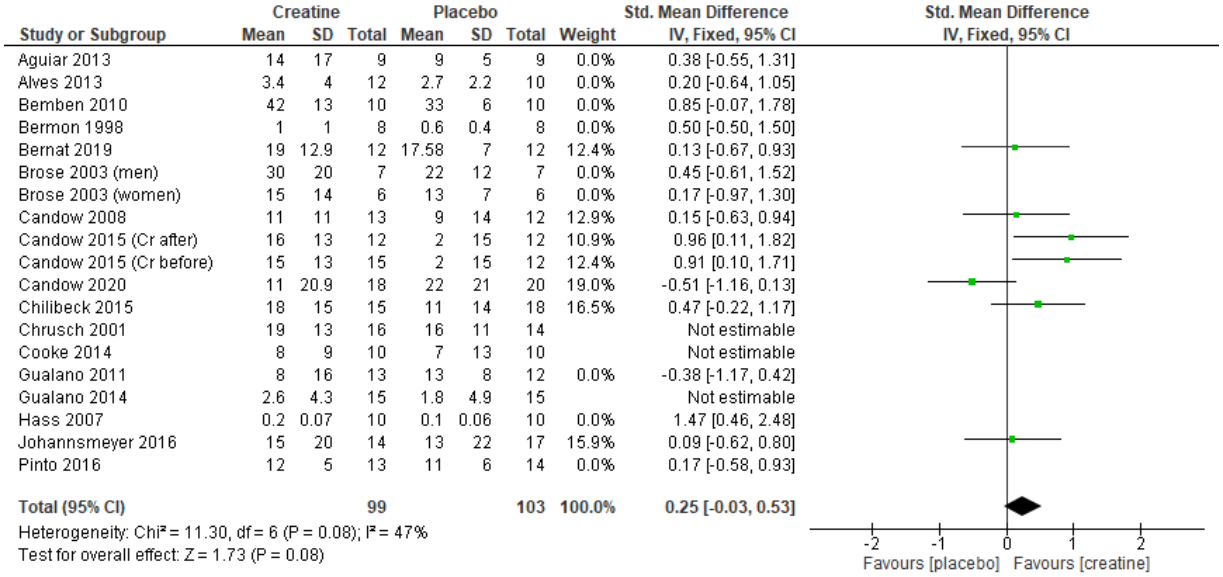

Figure S3: Forest plot for chest press strength with the inclusion of only high dose studies that did not include a creatine loading phase.

Study or Subgroup	Creatine Mean	Creatine SD	Creatine Total	Placebo Mean	Placebo SD	Placebo Total	Weight	Std. Mean Difference IV, Fixed, 95% CI
Aguiar 2013	14	17	9	9	5	9	0.0%	0.38 [-0.55, 1.31]
Alves 2013	3.4	4	12	2.7	2.2	10	0.0%	0.20 [-0.64, 1.05]
Bemben 2010	42	13	10	33	6	10	0.0%	0.85 [-0.07, 1.78]
Bermon 1998	1	1	8	0.6	0.4	8	0.0%	0.50 [-0.50, 1.50]
Bernat 2019	19	12.9	12	17.58	7	12	11.6%	0.13 [-0.67, 0.93]
Brose 2003 (men)	30	20	7	22	12	7	0.0%	0.45 [-0.61, 1.52]
Brose 2003 (women)	15	14	6	13	7	6	0.0%	0.17 [-0.97, 1.30]
Candow 2008	11	11	13	9	14	12	12.1%	0.15 [-0.63, 0.94]
Candow 2015 (Cr after)	16	13	12	2	15	12	10.2%	0.96 [0.11, 1.82]
Candow 2015 (Cr before)	15	13	15	2	15	12	11.6%	0.91 [0.10, 1.71]
Candow 2020	11	20.9	18	22	21	20	0.0%	-0.51 [-1.16, 0.13]
Chilibeck 2015	18	15	15	11	14	18	15.4%	0.47 [-0.22, 1.17]
Chrusch 2001	19	13	16	16	11	14	14.4%	0.24 [-0.48, 0.96]
Cooke 2014	8	9	10	7	13	10	9.7%	0.09 [-0.79, 0.96]
Gualano 2011	8	16	13	13	8	12	0.0%	-0.38 [-1.17, 0.42]
Gualano 2014	2.6	4.3	15	1.8	4.9	15	0.0%	0.17 [-0.55, 0.89]
Hass 2007	0.2	0.07	10	0.1	0.06	10	0.0%	1.47 [0.46, 2.48]
Johannsmeyer 2016	15	20	14	13	22	17	14.9%	0.09 [-0.62, 0.80]
Pinto 2016	12	5	13	11	6	14	0.0%	0.17 [-0.58, 0.93]
**Total (95% CI)**			**107**			**107**	**100.0%**	**0.37 [0.09, 0.64]**

Heterogeneity: Chi² = 5.39, df = 7 (P = 0.61); I² = 0%
Test for overall effect: Z = 2.63 (P = 0.008)

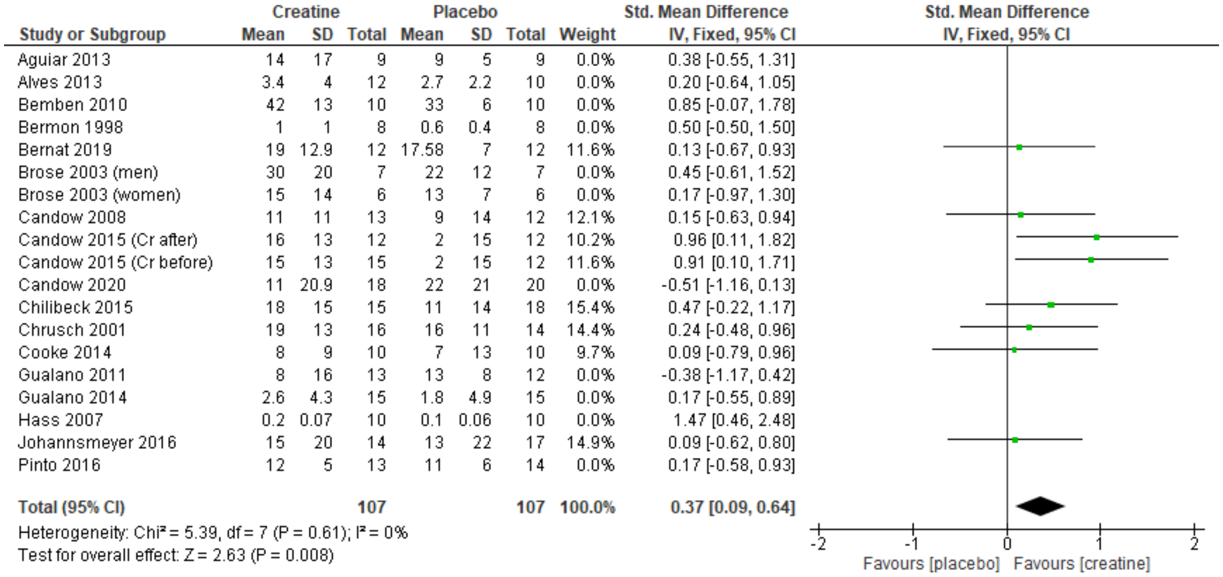

Figure S4: Forest plot for chest muscle strength with the inclusion of only high dose studies (> 5 g/day) with a sensitivity analysis showing that Candow et al. 2020 exclusion significantly altered the findings.

Study or Subgroup	Creatine Mean	Creatine SD	Creatine Total	Placebo Mean	Placebo SD	Placebo Total	Weight	Std. Mean Difference IV, Fixed, 95% CI
Aguiar 2013	14	17	9	9	5	9	0.0%	0.38 [-0.55, 1.31]
Alves 2013	3.4	4	12	2.7	2.2	10	0.0%	0.20 [-0.64, 1.05]
Bemben 2010	42	13	10	33	6	10	0.0%	0.85 [-0.07, 1.78]
Bermon 1998	1	1	8	0.6	0.4	8	0.0%	0.50 [-0.50, 1.50]
Bernat 2019	19	12.9	12	17.58	7	12	15.3%	0.13 [-0.67, 0.93]
Brose 2003 (men)	30	20	7	22	12	7	0.0%	0.45 [-0.61, 1.52]
Brose 2003 (women)	15	14	6	13	7	6	0.0%	0.17 [-0.97, 1.30]
Candow 2008	11	11	13	9	14	12	15.9%	0.15 [-0.63, 0.94]
Candow 2015 (Cr after)	16	13	12	2	15	12	13.5%	0.96 [0.11, 1.82]
Candow 2015 (Cr before)	15	13	15	2	15	12	15.3%	0.91 [0.10, 1.71]
Candow 2020	11	20.9	18	22	21	20	0.0%	-0.51 [-1.16, 0.13]
Chilibeck 2015	18	15	15	11	14	18	20.3%	0.47 [-0.22, 1.17]
Chrusch 2001	19	13	16	16	11	14		Not estimable
Cooke 2014	8	9	10	7	13	10		Not estimable
Gualano 2011	8	16	13	13	8	12	0.0%	-0.38 [-1.17, 0.42]
Gualano 2014	2.6	4.3	15	1.8	4.9	15		Not estimable
Hass 2007	0.2	0.07	10	0.1	0.06	10	0.0%	1.47 [0.46, 2.48]
Johannsmeyer 2016	15	20	14	13	22	17	19.6%	0.09 [-0.62, 0.80]
Pinto 2016	12	5	13	11	6	14	0.0%	0.17 [-0.58, 0.93]
**Total (95% CI)**			**81**			**83**	**100.0%**	**0.43 [0.11, 0.74]**

Heterogeneity: Chi² = 4.74, df = 5 (P = 0.45); I² = 0%
Test for overall effect: Z = 2.67 (P = 0.008)

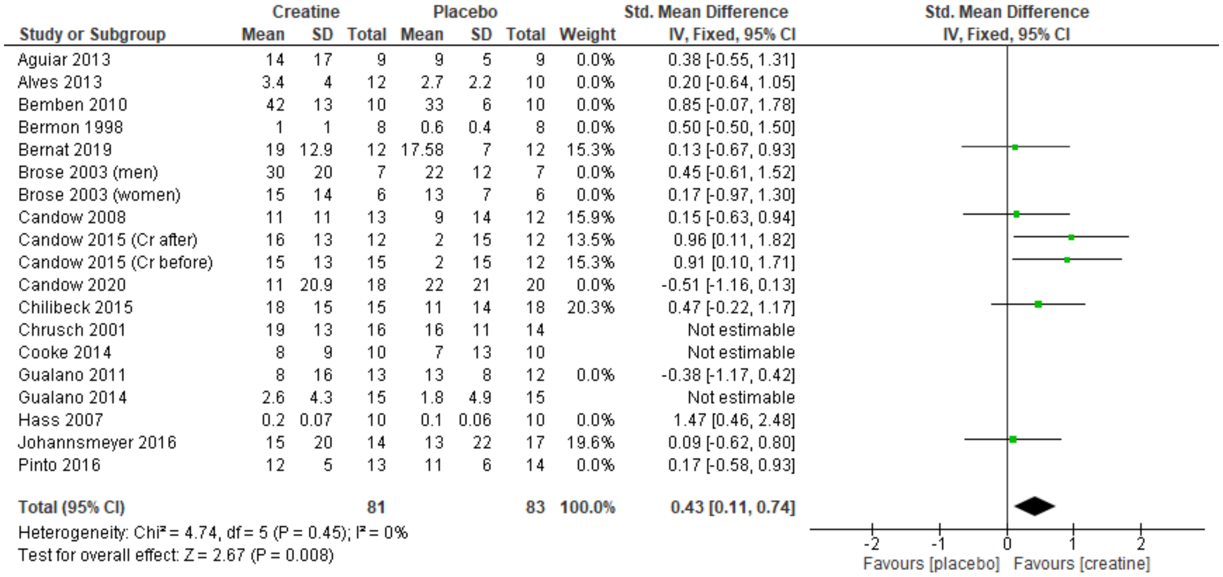

Figure S5: Forest plot for chest muscle strength with the inclusion of only high dose studies (> 5 g/day) that did not include a loading phase with a sensitivity analysis showing that Candow et al. 2020 exclusion significantly altered the findings.

Study or Subgroup	Creatine Mean	Creatine SD	Creatine Total	Placebo Mean	Placebo SD	Placebo Total	Weight	Std. Mean Difference IV, Fixed, 95% CI
Alves 2013	17	14	12	8	9	10	20.9%	0.72 [-0.15, 1.59]
Bemben 2010	40	16	10	33	32	10	0.0%	0.27 [-0.62, 1.15]
Bernat 2019	144.5	96.1	12	83.91	54.6	12	0.0%	0.75 [-0.08, 1.58]
Brose 2003 (men)	50	33	7	65	23	7	13.9%	-0.49 [-1.56, 0.58]
Brose 2003 (women)	42	36	6	47	18	6	12.3%	-0.16 [-1.30, 0.97]
Candow 2008	20	18	13	21	17	12	0.0%	-0.06 [-0.84, 0.73]
Candow 2015 (Cr after)	41	38	12	6	35	12	0.0%	0.93 [0.08, 1.77]
Candow 2015 (Cr before)	37	27	15	6	35	12	0.0%	0.98 [0.17, 1.79]
Candow 2020	69	35.6	18	76	38.1	20	0.0%	-0.19 [-0.82, 0.45]
Chilibeck 2015	54	49	15	50	45	18	0.0%	0.08 [-0.60, 0.77]
Chrusch 2001	50	24	16	29	19	14	0.0%	0.94 [0.18, 1.70]
Cooke 2014	67	46	10	64	52	10	0.0%	0.06 [-0.82, 0.94]
Gualano 2011	14	16	13	13	8	12	25.7%	0.08 [-0.71, 0.86]
Gualano 2014	14	13	15	10	9	15	0.0%	0.35 [-0.37, 1.07]
Johannsmeyer 2016	28	23	14	36	24	17	0.0%	-0.33 [-1.04, 0.38]
Neves 2011	10	11	13	11	11	11	0.0%	-0.09 [-0.89, 0.72]
Pinto 2016	54	47	13	71	33	14	27.2%	-0.41 [-1.17, 0.36]
**Total (95% CI)**			**51**			**49**	**100.0%**	**-0.03 [-0.43, 0.37]**

Heterogeneity: Chi² = 4.64, df = 4 (P = 0.33); I² = 14%
Test for overall effect: Z = 0.15 (P = 0.88)

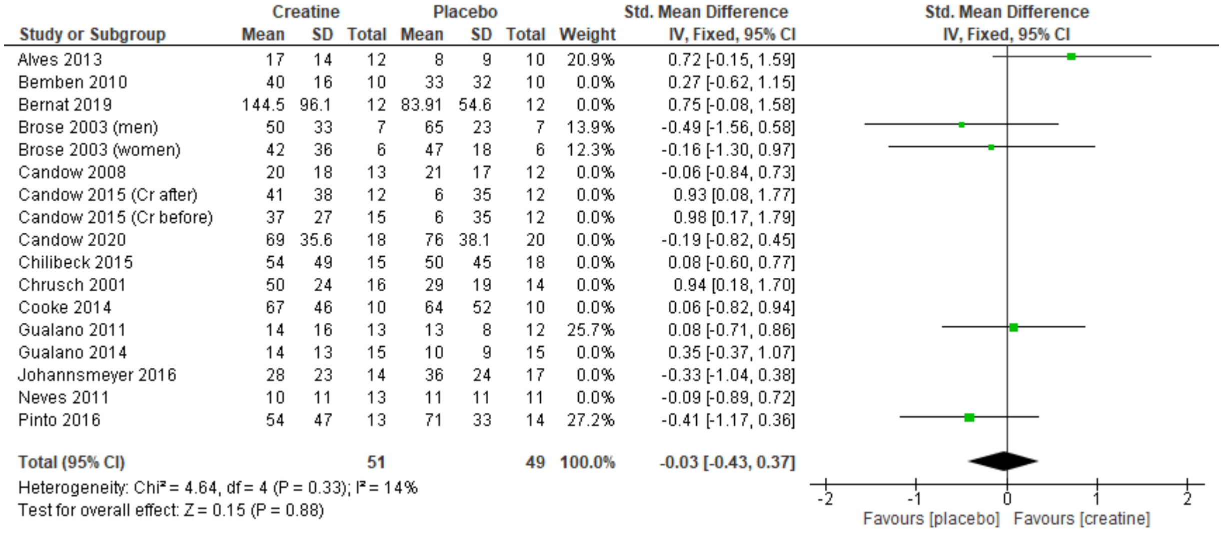

Figure S6: Forest plot for leg press strength with the inclusion of only low dose studies (≤ 5 g/day) that did not include a creatine loading phase.
